# Enabling Virtual Learning for Biomechanics of Tooth Movement: A Modified Nominal Group Technique

**DOI:** 10.3390/dj11020053

**Published:** 2023-02-17

**Authors:** Fakhitah Ridzuan, Gururajaprasad Kaggal Lakshmana Rao, Rohaya Megat Abdul Wahab, Maryati Md Dasor, Norehan Mokhtar

**Affiliations:** 1 Dental Simulation and Virtual Learning Research Excellence Consortium, Department of Dental Science, Advanced Medical and Dental Institute, Universiti Sains Malaysia, Bertam, Kepala Batas 13200, Malaysia; 2 Department of Orthodontics, Penang International Dental College, NB Tower 5050, Jalan Bagan Luar, Butterworth 12000, Malaysia; 3 Orthodontic Discipline, Department of Family Oral Health, Faculty of Dentistry, Universiti Kebangsaan Malaysia, Jalan Raja Muda Abdul Aziz, Kuala Lumpur 50300, Malaysia; 4 Centre of Pediatric Dentistry and Orthodontic Studies, Faculty of Dentistry, Universiti Teknologi MARA, Sungai Buloh Campus, Jalan Hospital, Sungai Buloh 47000, Malaysia

**Keywords:** dental education, orthodontic education, virtual learning, nominal group technique

## Abstract

Virtual learning is a medium that can enhance students’ understanding of a specific topic. The emergence of the COVID-19 pandemic provided an opportunity for dental education to shift from traditional learning to blended learning as it began to utilize technology to help students study effectively. In this study, we collaborated with experts in the field of dentistry to reach a consensus about which topics are appropriate to include in the virtual learning module about the biomechanics of tooth movement. We convened a panel of five experts who had a minimum of two years of experience in teaching orthodontics and introduced them to the Nominal Group Technique (NGT), which is a well-established, organized, multistep, assisted group meeting technique for generating consensus. The following ten key topics were identified for inclusion in the module: physiology of tooth movement; tooth movement–definition, type, theory, indications; force systems; anchorage; fixed appliances; biomaterials related to tooth movement; removable appliances; factors affecting tooth movement; iatrogenic effect of tooth movement; and current advances and evidence regarding tooth movement. The modified NGT approach led to the development of a ranked thematic list of the topics related to the biomechanics of tooth movement that can be delivered to students via virtual learning.

## 1. Introduction

Almost all students worldwide have been impacted by the COVID-19 pandemic, which has had an unprecedented and extensive effect on education [1,2]. Education systems around the world have faced major changes as schools and universities shut down, and educators have made significant efforts to adapt and innovate. In Malaysia, a movement control order was instituted in March 2019. However, Malaysia was already committed to making changes to develop global online learning, as stated in the Malaysian Higher Education Development Plan 2015–2025 [3]. The goals of these changes to the education system are to provide high-quality content, enhance the quality of teaching and learning, and reduce the delivery cost.

Since COVID-19 hit the world, online learning is no longer just an option, it is a necessity. More than 95% of all countries were affected by the presence of COVID-19 [4]. Face-to-face classes were temporarily suspended, and teaching and learning processes were moved to 100% online learning. This pandemic has revealed the opportunity to accelerate digital transformation in education, including medical education [5]. Even beyond COVID-19, online learning could have a favourable impact on future dental education.

The Nominal Group Technique (NGT) has been widely used in dentistry and dental education. Pahinis et al. [6] used it to evaluate a blended learning course taught to different groups of learners in a dental school. They used the NGT to identify strengths and weaknesses in a group setting that allowed participants to think of solutions and analyze and plan for their implementation. Tomlinson et al. [7] utilized the NGT to design the oral mucositis assessment instrument for use in children, and Hsu et al. [8] used it to identify the core competencies required by dental students at the time of graduation.

Currently, dental education emphasizes a pedagogical and apprenticeship approach, which is facing numerous issues that affect knowledge delivery and training [9]. Clinical skills are often taught to students through demonstrations on patients or in a simulated environment. Close observation, motor coordination, and desire to progress are some of the skills required to gain competency in these clinical skills. To achieve a specific level of proficiency, the practical tasks are typically repeated because not all students have the same level of hands-on experience. In the simulation lab, students must constantly reflect on their work and receive feedback in order to move to the next procedure. However, the feedback is usually provided by the tutor upon the completion of the task, before proceeding to the next task [10].

Virtual learning refers to technology-assisted teaching and learning for the transmission of knowledge. It has been defined in a variety of ways, but a recurring theme is an integrated set of elements that allow students and teachers to engage in ‘online’ activities, including online learning [11]. The current education methods utilize e-learning approaches in the delivery of content. E-learning is one aspect of the virtual learning environment, which consists of web-based learning, online learning, computer-assisted instruction, internet-based learning, and distance learning [12]. E-learning could be a good alternative to traditional learning for providing high-quality education [13]. However, there are few evidence-based findings to guide future online learning of the pedagogical components of dental education, as face-to-face classroom lectures in a didactic format are significantly used in teaching and learning.

The acceptance of technology in healthcare practise and services in working toward Industry 4.0 has been demonstrated, but this technological swiftness has not been seen in educational procedures. Specialty orthodontic training utilizes current technological advances to understand the biomechanics of tooth movement, but the acceptance and use of technology in orthodontic student education is currently limited and poorly designed. In a fixed preclinical laboratory setting, the didactic technique with two-dimensional learning resources is still used in teaching and learning cycles [14].

Virtual learning supports teaching and learning by providing e-learning resources online and encouraging participation in interactive elements such as assessments, assignments, and discussions [15]. The main advantage of virtual learning is that the learners can access the instructional content at any time and from any location [16]. A study of virtual learning in other faculties of dentistry, such as the radiological interpretation of bony lesions of the jaw, revealed superior learning and improvements in knowledge acquisition compared to in-class learning [16]. The “Virtual Dental Implant Training Simulation Program” was created by The Medical College of Georgia School of Dentistry to help to improve students’ understanding of diagnosis, decision making, and treatment protocols [17]. These studies show that the development of virtual learning in orthodontic education is critical.

According to a survey conducted by Poblete et al. [18], 70% of the dental students and academics who participated ranked the topic of the biomechanics of tooth movement as a beneficial item in three-dimensional digital resources. Learning about the biomechanics of tooth movement can be difficult because the concepts are hard to grasp in a static context [19]. However, the animations and feedback provided by an interactive medium dramatically improve the understanding of the relationships between force application and tooth movement. The principles of biomechanics have not changed, but computer technology has evolved significantly in the last 14 years, and higher graphic display standards have resulted in significant improvements in visual and audio presentations [20]. The goal of this study was to determine which topics related to the biomechanics of tooth movement are appropriate for inclusion in a virtual learning module that could be implemented and delivered to dental students.

## 2. Methods

Ethical approval for this study was granted by the Human Research Ethics Committee of Universiti Sains Malaysia (USM/JEPeM/21110756).

### 2.1. Study Population and Sampling

Orthodontic experts were invited to participate in this study. The selection criteria were based on Dalkey and Helmer [21]. Swanson and Holton [22] defined experts as those who are knowledgeable and skilled in a field of study. The expert panel in this study consisted of lecturers with a minimum of two years of experience in teaching orthodontics and with a deep knowledge and understanding of the context of the study. Another criterion for participation in the study was digital literacy.

Van De et al. [23] stated that between five and nine experts should be involved in collecting data using the NGT approach. Thus, we recruited five experts to participate in this study through a targeted invitation to three Malaysian dental schools (Universiti Sains Malaysia (USM), Universiti Kebangsaan Malaysia (UKM), and Universiti Teknologi Mara (UiTM)).

### 2.2. Design

In this study, we adapted the NGT to identify the topics suitable for virtual learning that would help increase student knowledge. The NGT methodology, which was originally developed by Van de Ven and Delbecq [23] in 1971, generates more ideas than traditional group discussions, while reducing competitiveness and pressure to conform based on status within the group. We chose this technique as the methodology for achieving consensus when participants contribute to and develop ideas about an issue [24]. The five advantages of the NGT approach are as follows [25]:

1. It can balance a participant’s rank and educational background and allow them to speak based on their experience and knowledge of some issues.
2. The technique is used in a group setting, allowing the participants to focus on an issue.
3. Each participant is given the opportunity to turn their idea into a brief note.
4. The technique allows users to record their ideas.
5. Issues can be thoroughly addressed to prevent confusion among participants in the study.

The NGT shares several features with the Delphi group consensus method; however, in contrast to the Delphi group consensus method, it is a structured face-to-face interaction that usually involves 5–12 participants [26]. Various modified NGT methods have been established to overcome this limitation. For example, Kottman et al. [27] conducted a NGT process that consisted of three rounds of emails prior to the participants’ consensus meeting. The first email message asked the experts to rate the importance of quality indicators, and the participants were allowed to propose three to five new indicators. In the second and third rounds, the experts were asked to rank the quality indicators and propose modifications if required. Finally, the consensus meeting was held to evaluate, revise, and agree on the quality indicators. The drawback of this modified NGT approach was a poor response rate from participants.

Kelly et al. [28] used focus group discussion together with the NGT to explore the reasons for participants’ choices after the preliminary vote process. In contrast, O’Grady et al. [29] excluded the preliminary voting stage. They claimed that the fifth stage (voting and ranking) of the NGT was not meaningful for understanding how oral health academics conceptualize health promotion and perceive the barriers and possible opportunities for health promotion implementation in dental practice.

Duffy et al. [30] conducted the NGT as a half-day consensus development meeting. Before the meeting, the participants provided their demographic details and made an explicit commitment to participate actively. Following an initial discussion, the outcomes were divided into three provisional categories: (1) outcomes to be considered for inclusion in the final core outcome set; (2) outcomes where no consensus existed; and (3) outcomes that should not be considered for inclusion in the final core outcome set. The participants discussed the ordering of the outcomes within each category and the outcomes could be moved between the categories.

**Figure 1:**
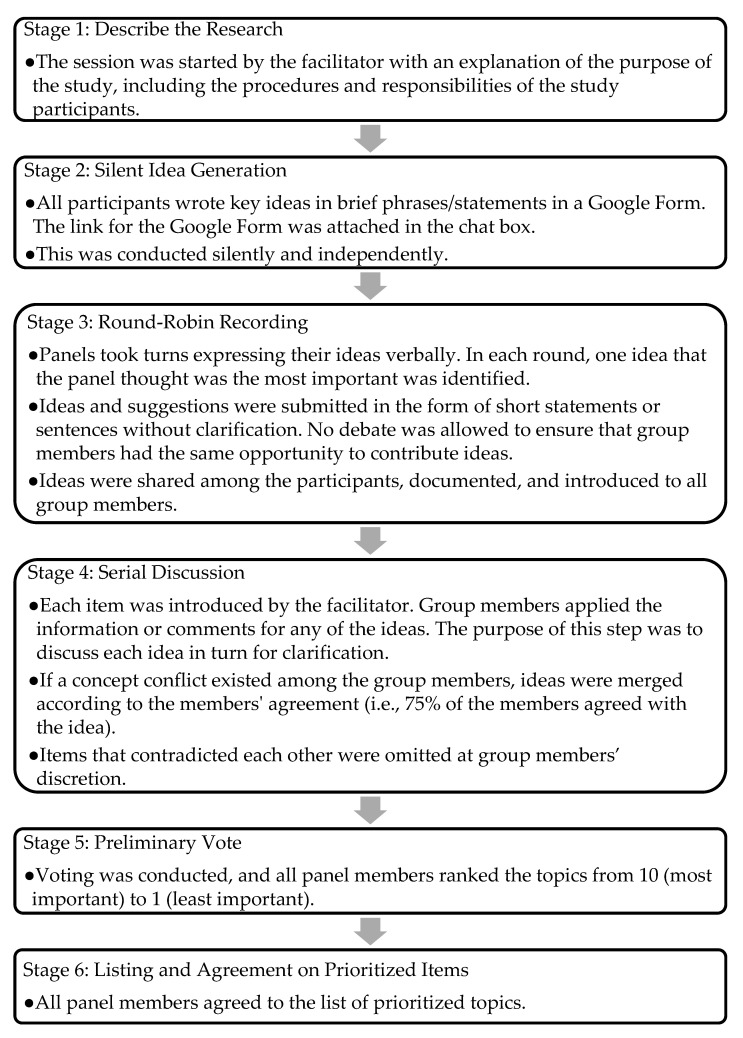
Steps in the NGT approach used in this study.

In this study, the NGT meeting was conducted virtually through Webex due to the restriction of movement order in Malaysia in response to COVID-19. Figure 1 shows the steps used during the sessions. Before the meeting, an email was sent to all expert members so that they could agree on a suitable date and time. All six steps were implemented, and technologies such as Google Forms and Microsoft PowerPoint were used to facilitate the consensus meeting.

## 3. Results

### 3.1. Consensus Process

The expert consensus meeting was successfully conducted online to achieve the objective. The meeting lasted approximately 3 h and 15 min. Even though it was conducted virtually, this modified NGT was able to provide almost the same atmosphere as the original in-person NGT. All of the experts were able to complete each phase of the NGT without any problems. The use of various technologies, such as Google Form to provide ideas and PowerPoint to serve as a whiteboard to record ideas and make summaries, was very helpful during the session. As Webex sessions can be recorded, the facilitator could easily review the recording for reporting purposes.

### 3.2. Virtual Learning about Biomechanics of Tooth Movement 

The consensus meeting started with an introduction to the NGT, including the procedure and role of each panel member. Each participant was given the same opportunity to voice ideas at each stage of the process. Forty-one ideas were generated during the silent generation stage from the input provided by participants on the Google Form. Next, the panel members were asked to express their ideas verbally in short statements and without clarifications. After this phase, only 27 ideas remained because 34% of the initial ideas overlapped. During the serial discussion stage, the 27 ideas were grouped into similar topics based on the participants’ agreement. None of the items contradicted one another, and none were omitted from the list of ideas. All of the ideas were critically discussed among the experts and, ultimately, ten topics were identified. 

In the preliminary voting process, each participant was asked to rank the topics that they personally felt were the most important and to write them down, in order of priority, on a sheet of paper [31]. The highest priority was assigned a score of 10, and the lowest priority was assigned a score of 1. Based on the NGT results, the ten topics were ranked in order of their priority for implementation in the virtual learning system. Table 1 shows the topics and subtopics prioritized during the NGT meeting in response to the question: What are the topics in the biomechanics of tooth movement that can be included in the virtual learning module?

## 4. Discussion

### 4.1. The Modified NGT 

Although we describe our method as a modified NGT, all of the stages were the same as those of the original NGT. The only difference was the virtual medium used. This modified NGT allowed us to invite experts from different locations to participate in the study. Conducting the study virtually made it easy to plan the consensus meeting because the experts did not need to be physically present in one place. In addition, the recording feature of Webex allowed all of the ideas proposed by the participants to be further documented, making the NGT process easily manageable. However, the limitation of this modified NGT was that what was being discussed was not always clear due to poor internet connections. 

### 4.2. Virtual Learning about Biomechanics of Tooth Movement 

Virtual learning programs have received a lot of attention in recent years, and they emerged quickly due the COVID-19 pandemic. However, converting from traditional, in-person learning to virtual learning is not an easy task. In the field of dentistry, it requires the identification of which topics are suitable for online delivery, as dental education consists of theoretical and clinical sessions. The goal of this study was to identify the topics that are suitable for virtual delivery, and we focused on the biomechanics of tooth movement. We used the NGT technique, which is a very effective and efficient method for gathering consensus on a complicated issue.

The experts provided some general comments about the limitations of in-class learning. For example, they stated that the limit of a three hour session is not enough to teach a topic. Inquimbert et al. [32] reported that virtual learning was required for pedagogical support to assist in the understanding of lectures and practical work. Virtual learning education also can be applied to preclinical dental education, based on the student’s ability and learning styles [33].

The biomechanics of tooth movement is a critical subject in the orthodontic curriculum because most of the topics require visualization. The teeth move inside the bone, and this process is difficult to explain without visual tools. At present, students learn the topics theoretically, but simulations could help them visualize tooth movement to achieve a clear understanding of how the teeth move inside the bone. Video simulations could be used for this purpose. Video is a component of virtual learning that appeals to students and helps them understand lectures and clinical practice [32]. They also feel that virtual learning is very useful for summarizing and enhancing comprehension, which cannot be achieved in traditional classes [32].

In this study, the topic most highly ranked by the experts was the physiology of tooth movement, which includes periodontal and bone responses to normal function and orthodontic force. When this topic is delivered to the students, they need to understand what happens at the cellular level during the normal function of tooth movement. Therefore, delivering this information virtually should improve students’ understanding because they can see how the movement of teeth occurs and how the cells react. Hakami [34] reported that through virtual learning, students were able to keep up with the latest medical advances due to open-access guidance from medical experts, and they were able to retrieve the knowledge lost due to the suspension of classes and clinical postings because of COVID-19.

Typically, the teaching of theory, such as the introduction to tooth movement, which includes definition, type, theory, and indication of orthodontic tooth movement, is delivered to students through lectures. However, by using the various types of media available in virtual learning, students can take advantage of the available features to better understand the theory. Liebermann and Erdelt [35] reported that 92% of the students in their study said that they could understand dental morphologies better using virtual learning compared to using a typical textbook [35]. Soltanimehr et al. [16] showed that virtual learning outperformed the traditional lecture-based method for enhancing knowledge acquisition in the radiographic interpretation of bone lesions of the jaw, and Hakami [34] found that student achievement in virtual learning was as high as it was in a traditional face-to-face classroom. Thus, virtual learning exhibits a positive trend of increasing theoretical knowledge [36].

The moment of force and mechanical principles in orthodontic force control, such as one-couple vs. two-couple systems, are also important topics that need to be delivered in the virtual learning system. The amount of movement of the posterior teeth (molars and premolars) to close the extraction space is known as anchorage [37]. As anchorage necessitates a customized treatment strategy that can range between no permitted mesial/premolar movement (or distal movement of the molars may even be required) and complete space closure by protraction of the posterior teeth, it is easier for students to learn about anchorage virtually. They can experiment on their own to visualize the amount of force and movement needed for different cases.

Although the knowledge delivered by a lecturer is very dynamic, using virtual learning allows students to learn about case studies on their own and understand more about the treatment plan that can be developed for patients. Virtual learning also allows students to predict the result based on the treatment plan, which would be helpful for students when preparing for their clinical years. The aim of the virtual learning described herein is to complement traditional learning, because the blending of traditional face-to-face and virtual learning is the best teaching and learning approach [38]. Currently, blended learning simply utilizes e-learning as the learning platform and includes theoretical knowledge delivery and demonstration. Virtual learning, which includes simulation technology, is relatively inexpensive and safe [39], and it allows students to work on difficult problems and apply time-consuming techniques for evaluating patient samples.

## 5. Conclusions

We used a modified NGT to gather opinions and consensus about which topics related to the biomechanics of tooth movement can be delivered virtually to dental students. The modified NGT allowed for greater expert participation compared to a face-to-face meeting because it was held online. The ten elements on the list created in this study should lead to much-needed discussion about curriculum reform in the virtual learning environment for orthodontic education. Virtual learning will serve as an essential tool for the delivery of content related to the biomechanics of tooth movement because theoretical content can be transformed into visualizations that improve students’ understanding of the topic. The list of prioritized topics produced by the experts can be used to help curriculum designers develop virtual learning tools to support and improve students’ understanding and knowledge acquisition. We conclude that the NGT successfully helped us meet the objectives of this study by identifying and exploring the relevant topics to be presented virtually.

## Figures and Tables

**Table 1:** Priority topics identified by the expert panel via the NGT.

Ranking	Topics	Subtopics
1	Physiology of tooth movement	Periodontal and bone response to normal function and orthodontic force Reactions of cellular
2	Tooth movement-definition, type, theory, indications	Definition of tooth movement Types of tooth movement Theory of orthodontic tooth movement Indications of tooth movement
3	Force systems	Moment of force Force systems (equal, couple, single) Forces delivered by orthodontic appliances (continuous, intermittent, interrupted) Mechanical principles in orthodontic force control: one-couple vs. two-couple systems
4	Anchorage	Anchorage and its control; anchorage considerations Temporary anchorage devices Adjunct appliances such as mini-implants, expansion appliances, headgears, protraction facemask
5	Fixed appliances	Fixed appliance treatment mechanics: Stages 1, 2, and 3 General using fixed appliances, as in bodily movement, rotation, expansion, distalization general overview Biomechanics involved in aligning impacted teeth such as impacted canine, incisors, or premolars; their path of eruption; mechanics of fixed appliances, vectors, and forces involved Interceptive and preventive procedures
6	Biomaterials related to tooth movement	Biomaterials and production of orthodontic forces Basic principle of elastic and archwire materials
7	Removable appliances	Interceptive and preventive procedures Removable appliances: overview
8	Factors affecting tooth movement	Factors affecting tooth movement Patients’ factor to orthodontic tooth movement Comparison of biomechanics in terms of growing and non-growing, with regards to removable and fixed appliances Limitations
9	Iatrogenic effect of tooth movement	Iatrogenic effect of tooth movement Problems halting tooth movement in orthodontics
10	Current advances and evidence regarding tooth movement	Studies or research related to biomechanics in orthodontic tooth movement: advances in orthodontic research

## Data Availability

Not applicable.
